# Folktales as indigenous pedagogic tools for educating school children: A mixed methods study among the Nso of Cameroon

**DOI:** 10.3389/fpsyg.2023.1049691

**Published:** 2023-02-06

**Authors:** Lilian F. Wiysahnyuy, Ngalim Banfegha Valentine

**Affiliations:** ^1^Department of Guidance and Counselling, Higher Teacher Training College, The University of Bamenda, Bamenda, Cameroon; ^2^Department of Philosophy, Higher Teacher Training College, The University of Bamenda, Bamenda, Cameroon

**Keywords:** culture, indigenous pedagogy, informal learning, folktales, teachers, parents, elementary school

## Abstract

In many low and middle-income countries, schools are sites of cross-cultural contact and conflict due to a mismatch between the learning priorities and pedagogical practices used in children’s homes and those they encounter in school. The alien nature of school as a learning context contributes to several negative outcomes. It hampers children’s acquisition of academic skills, limits their parents’ engagement in their schooling and prevents schools from benefiting from the learning resources and pedagogical practices available in the local context. To prevent this, many low and middle-income countries are considering the feasibility of incorporating local cultural activities into the school curriculum as a means of bridging the cultural divide between schools and the communities they serve. This research aims to inform these decisions through a case study of stakeholders’ views towards the integration of a specific indigenous pedagogic tool, folktales into school settings in Cameroon, Central Africa. Qualitative data were gathered from 20 teachers and 15 parents in Kumbo, the capital of the Nso Kingdom where traditional child-rearing customs remain strong. A thematic analysis of the data revealed that most parents told their children folktales as one component in an indigenous participatory pedagogy designed to stimulates some cognitive skills and enhance moral development among children. Most teachers and parents believed that there were many potential importance of integrating folktales into schools as a traditional pedagogy for schoolteachers’ professional practice. All teachers reported that they used folktales during lessons and/or pupils’ free time. Like parents, teachers both narrated folktales and invited children to narrate them though some parents indicated that it was not necessary. This pattern of findings supports the conclusion that folktales provide invaluable pedagogic values and prospects for schools and formal education in general. Caution should be exercised in any attempt to generalize these findings because the sample and the geographical site were limited. However, this study suggests that folktales may be a readily available indigenous pedagogic tool that has both a high level of acceptability among teachers and parents and can be effective in bridging the cultural divide between schools and communities in many low and middle-income countries.

## 1. Introduction

Although it is a relatively recent development, today there is almost universal acknowledgement of the importance of formal schooling as an agent of education in low-and middle income countries. Previously, most education was organized informally through agencies such as the home, community and peer groups. Regardless of the questionable motives of many who introduced formal schooling to these countries, one consequence of dramatic cultural and economic changes these countries have recently seen is that it is no more possible for young people to acquire the skills and knowledge necessary to capitalize on life opportunities and to fully participate as citizens for survival and for the good life solely on the basis of informal education as was the case in traditional society. Despite the important role that schools now play, traditional patterns of informal education have not been abandoned. For example, in communities across, African parents continue to inculcate indigenous knowledge in their children through the use of riddles, proverbs, folktales, traditional games, and songs (Omolewa, 2007; Ombola, 2013; Osaat and Asomeji, 2017). These pedagogical tools helped children retain what they learned, fostered creativity, and improved their problem-solving and language skills. These are goals shared by formal schooling, however, it is unclear whether schools incorporate these pedagogic tools as means to achieve them. Nevertheless, in many African countries there appear to be a sharp junction between the goals and pedagogies that characterize the education children receive in their families and communities and those that characterize the education children receive at school. Education is the process by which society deliberately and actively transmits its accumulated knowledge, skills and values from one generation to another. In the African context, traditional informal education was well conceived and pursued collectively for the good of the community. Families and communities continue to endorse the underlying aim of traditional African education, that is, to progressively connect children to their cultural heritage, ways of life and the continuation of their family and community (e.g., Gudhlanga and Makaudze, 2012; Motsei and Phindane, 2021). However, in many African countries, school systems do not consider cultural transmission to be part of their educative responsibilities (Ngalim, 2014a,b).

In most African countries, schooling continues to be influenced by the models of Western education that were introduced by European powers primarily to train civil servants to support their colonial administrations. The Africanization of schools was a priority for most newly independent African countries during the 1960. This process involved a critical appraisal of the goals, curricula, pedagogies and assessment practices of the formal education systems they had inherited (Eisemon et al., 1986). Nonetheless, long after the end of colonial rule, the main focus of many African school systems remains relatively unchanged and schools fail to capitalize on the strengths of traditional African pedagogies (Shizha, 2013). Many of these allow room for critical thinking, capture children’s interest in further learning and encourage interaction between teachers and learners and between learners, thus increasing high level of participation. This traditional method of teaching enables children to store up images gained through observation, interaction, and oral narratives (Pence and Nsamenang, 2008).

Folktales are a staple of informal education across traditional societies in Africa (Sone, 2018; Agwuele and Nyamwange, 2021). They are stories that have been passed down from generation to generation. Folktales generally have a simple plot, involving characters that serve as symbols for different kinds of people. These characters encounter situations and events that have relevance in the lives of audience members, even though they are often unrealistic (e.g., talking animals; Adeyemi, 2021). Bascom (1954) proposed that folktales serve four functions: amusement; validation of culture by justifying its rituals and institutions; as a medium for moral education; and a nudge to maintain conformity to accepted patterns of behavior. Because specific folktales are likely to focus on only a subset of these functions, they can be classified into categories. Three common categories of African folktales are moral tales, in which good behavior is rewarded and bad behavior is punished (Paul, 1992); aetiological tales, which explain why things are the way they are (Paul, 1992); and dilemma tales, which pose problems that need to be resolved (Achufusi, 1986).

Although written text has existed in Africa for many centuries (Ngom, 2018), most was in Arabic. Because most of the population was not literate in Arabic, the transmission of folktales was almost exclusively oral. Thus, folktales have been described as, the prose narrative genre of oral literature. The long oral tradition of African folktales invests them with distinctive characteristics. First, narrating an African folktale is a creative process. Gifted storytellers adapt the text of the tale to make it more interesting and meaningful to specific audiences, including children (Bascom, 1954). Second, in many traditions, folktales include a range of literary genres, including not only narrative prose, but also poetry and songs (Ugochukwu, 2005). Third, African folktales are not monologues. They are told in suspenseful, engaging ways, and require the audience to pay attention so that they are ready to follow instructions, as the audience is usually required to make responses (e.g., through antiphony or call-and-response; Motsei and Phindane, 2021; Hayashi et al., 2022). In many African traditions, the audience may also interrupt the performance of a folktale in order to make comments (Essegbey, 2021). Fourth, by participating in the story-telling and subsequent meaning-making discussions, audience members are guided to perceive relationships between the imagined situations narrated in the folktale and the realities in their own environment (Jirata, 2018). These characteristics allow African folktales to deliver “didacticism without sermonization” (Afolayan, 2021).

Because they have these characteristics, it has been argued that folktales are among “the most readily available but effective pedagogical tools a teacher could utilize in the teaching of any subject, at all levels and in all settings” (Afolayan, 2021, p. 1001). Mweti (1999) proposed that the content of African folktales can be used to support pupil well-being in five ways: to build bridges between cultures through the shared tradition of storytelling; to engage pupils’ emotions; to help pupils to recognize the shared experiences and problems faced by people from different cultural backgrounds; to serve a cathartic effect by allowing the safe expression of emotions and to allow pupils to confront fears and solve problems “at one step removed”; and to help pupils to make sense of their worlds. Other authors have argued that storytelling is a pedagogic practice that supports the acquisition of a range of academic skills. These include enhancing pupils’ imagination and ability to visualize verbal descriptions, fostering their appreciation of the rhythm of language and increasing their vocabulary, enhancing speaking and listening skills, providing opportunities for critical thinking and creativity, revealing the ways in which literature can reflect and provide insights into human experience, and helping pupils to gain a deeper understanding of their own cultural heritage and that of others (Collins and Cooper, 2005; Wiysahnyuy, 2013, p. 188). In addition, many African folktales demonstrate the use of a range of literary devices such as similes, metaphor, imagery, repetition, personification, and hyperbole (Adeyemi, 2021) and a range of speech forms (e.g., commands and requests) and speech styles (e.g., formal and informal).

Although folktales continue to be valued across diverse childrearing contexts in Africa, cultural changes have led to changes in the agents transmitting folktales in some contexts. For example, among the Oku who inhabit the Cameroon Grasslands, adults were traditionally the narrators of folktales (Mbunwe-Samba, 1998). Argenti (2010) found that these are now almost exclusively transmitted from older to younger children, with no adult involvement. Oku children tell folktales around the hearth fire after the evening meal. One will announce a desire to tell a folktale and if other children are interested, they will signal this and fall silent. Once that child has told his or her folktale, and stated its moral, other children may follow, often resulting in protracted storytelling sessions. Although the shift in the role of narrator has preserved the salience of folktales in children’s lives, it may limit the extent to which continuity between formal and informal learning contexts can be increased by the adoption of folktales by schoolteachers.

Other communities in Cameroon, including the Nso, are also witnessing cultural change. Twenty-five years ago, Nsamenang and Lamb (1993) observed that in Nso communities, “the social system is in total flux… children are frequently more knowledgeable in matters of contemporary society than their…parents. This reverses traditional roles and makes it difficult for parents to be role models, little is known of the extent to which traditional values are being renounced in the process” (Nsamenang and Lamb, 1993, p. 433). The pace of social and economic change has increased since then. The participatory nature of traditional folktale performances is very consistent with Nso child-rearing philosophies. The Nso use a participatory, rather instructional, approach to child-rearing (Nsamenang et al., 2008). Nso children are cultural “agents” in their own learning. Adults believe them to be capable of learning from the educational precepts and practices that are embedded in family traditions, daily routines and social and communal activities, including the performance of folktales (Nsamenang et al., 2008). Nso children’s academic achievement is influenced by either the sharp disjunction between the participatory informal learning that occurs outside school and is supported by oral language in their mother tongue, Lamnso’, and the formal, instruction-based learning in English that occurs in school and is supported by written language (Trudell, 2006). Currently only 24.1% of children in Cameroon achieve at least a minimum level of proficiency in reading by the end of their elementary education and only 11.8% achieve at least a minimum level of proficiency in mathematics (UNESCO, 2017). Thus, Nso communities in Cameroon provide a useful context in which to explore folktales as a readily available resource with the potential to improve school learning.

The aim of the current study was to investigate whether a shared use of folktales as a pedagogic tool could serve as a bridge between children’s informal learning from adults in their homes and their formal learning from adults in schools. The first set of research questions therefore sought to establish whether Nso parents continue to use folktales in their informal learning practices; to identify the purposes for which these are used; and to explore the level of support among parents for teachers to use folktales as a pedagogic tool in their professional practice. The parallel second set of research questions investigated whether (and how often) elementary school teachers in Nso communities use folktales in their professional practice; the purposes for which these are used; and their level of support for folktales to become a standard pedagogic tool in teachers’ professional practice.

## 2. Methods

### 2.1. Context

The Bamenda Grassfields are large area of savannah occupying much of the Northwest Region in Cameroon. Nsoland is the largest kingdom in this region. Local political power remains within the royal dynasty. Although most Nso are Christian, a significant minority are Muslim or follow indigenous spiritual traditions (Nsamenang and Lamb, 1993). The province is part of Anglophone Cameroon, where the British colonial rule predominated. However, because the region lacked both resources and a strategic location of interest to European powers, the influence of colonial powers was weaker than in many other regions in Africa (Nsamenang and Lamb, 1995). There are ongoing political tensions between Anglophone Cameroon and the regions of Cameroon colonized by the French. These tensions frequently escalate into localized conflict, and disruption of the functions of civil society (World Bank, 2021). Both authors have a deep knowledge of Nso culture.

### 2.2. Participants

The participants of this study comprised Primary school teachers (*n* = 20: 60% male; age: 20–50 years) and parents (*n* = 15: 8 male; age; 20–55 years) drawn from three primary schools affiliated with religion (St. Theresia Primary school, Kumbo; Presbyterian School, Kumbo; and Cameroon Baptist Convention Primary School Kumbo). Fifty percent of teachers were selected from each school. Due to a political crisis, these were the only functioning primary schools in Kumbo central at the time of data collection. All government-run schools were closed for an extended period. All teachers who were available and willing to be interviewed were included in the sample (80% participation rate). Five parents whose children attended each school were recruited. It was necessary to use the snowball sampling technique because many parents were reluctant to invite unfamiliar adults to their homes during the conflict.

### 2.3. Interviews

Two interview guides were developed for the semi-structured interviews. The prompt questions in the interview for teachers focused on whether they used folktales in school and, if so, who narrates the folktales, and the learning contexts in which they used folktales. Regardless of whether the teacher had previously used folktales, the second part of the interview focused on teachers’ perceptions about the pedagogic potential of folktales in school learning tasks and how they could be used. The interview guide for the parents focused mainly on the types of folktales they tell their children, what children learn from them and parents’ perceptions about the pedagogic potential of folktales in school learning tasks. Although most questions in both interview guides were open-ended in order to elicit qualitative data, a small number of closed questions collected quantitative data. It should be noted that this study was purely qualitative, though a small amount of quantitative data was used to supplement the qualitative data.

### 2.4. Procedure

All interviews were completed during the same three-week period. To ensure informed consent, participants were given detailed information about the research and were assured of confidentiality. Interviews with teachers were carried out in schools; while interviews with parents were carried out in their homes. Each interview took about 30 min. The interview for teachers were conducted in the English language which is the first language in schools in Anglophone Cameroon. For the parents, seven of them were interviewed in English language while eight were interviewed in “lamnso” the local language of Nso. Both researchers were of Nso origin and could conduct the interviews in the two languages fluently.

### 2.5. Data analysis

For the analysis of data, this research followed five procedural steps proposed by Kvale (1996).

– Meaning condensation: this is also referred to as data reduction. It is the transformation of raw data into a form that can be analyzed. This has to do with developing units of meaning.
– Meaning categorization: This is the stage of coding. That is grouping the responses into categories that bring similar ideas, concepts and themes together. The emphasis is laid on uniformity.
– Meaning structuring. This has to do with comparison across themes that emerge so as to construct or remould the findings into a coherent story. There is the search for linkages across categories. This is stringing themes together, looking for over searching themes.
– Meaning interpretation: This is the meta-analysis of the emerging trends. It implies going “*beyond what is directly said to work out structures and relations of meaning not immediately apparent in a tex*t” (Kvale, 1996, p. 201) says it is putting the data “in the context of broader theory”.
– Meaning generation: This means systematizing the hermeneutic findings.
  In addition, the analysis of data was done following the systematic process of thematic and content analysis (Weber, 1990; Taylor-Powell and Renner, 2003) and narrative analysis (Propp, 1968).The first stage involved deciding on the level of analysis. At this level, single words, clauses and sets of words or phrases were coded. We decided on how many different concepts to code. This involved developing pre-defined or interactive sets of concepts categories. We had a code list earlier developed based on the major indicators of the study. The primary documents of textual data were coded for existence and for frequency of concepts by coding for every single positive or negative word or phrase that appeared. Relevant categories not included in the initial code list were added during the coding process. Introducing this coding flexibility allowed for new, important material to be incorporated into the coding process that could have significant bearings on results. During the coding it was assumed that any idea that emerges at least once is relevant. The ideas are therefore considered more important than frequency. However, the frequency also reflects how many times a concept emerges and is a major indicator of emphasis. The researcher coded ideas based on the participants responses which indicated whether they were neutral, for or against the idea.

## 3. Results

### 3.1. Parents’ use of folktales with their children

Qualitative data indicated that parents often narrated folktales to their children after dinner. At times they also asked their children to narrate the folktales to others. Most of the parents valued folktales for their focus on three themes that were important in their child-rearing: tradition/cultural values, wisdom and morality. For example, several parents indicated that folktales highlight the importance of wisdom in settling disputes or solving problems. In some cases, this theme was explicitly stated: “I tell my children folktales which teaches about wisdom. One of my most famous folktales is titled “*lim san kpu ee”* meaning the death of the rice farmer had a farm where bird used to eat everything in his rice farm. One day he killed an animal, removed the intestines and put on top of his stomach pretending that he was dead in order to catch the birds… and he succeeded. From that day his rice farm was safe. This shows how wise the rice farmer was.”

Similarly, some parents indicated that folktales promote morality as one stated: “I know that lies telling is a sin and I do not like my children to tell lies. One of the folktales I used to portray it is titled “*Ker Vifii e shaa Fon*” meaning wiser than the king. This folktale is about the tortoise which used to tell lies every day to the *fon* in order to gain favors… 1 day he was caught and locked up in the palace prison for deceiving the *fon*… He begged for forgiveness and after some days he was freed. He promised never to tell lies again. After these types of folktales, I always ask the children to tell me the lessons they have learned from them.”

Another folktale explained by parents’ revealed agricultural tradition and cultural beliefs of the people. The story revealed the deceptive character of a child who lied the parents about maize cultivation during the planting season. On the basis of this deception, he had to continue with tricks and deception during the harvesting season. This child lied his parents that in order to get a good harvest, one needs to prepare the maize with groundnuts prior to planting. As this was done, he went to the farm and ate all what was given to him as seeds. At harvest time, he had to fake a masquerade to frighten villagers from their farms in order to carry their products. He continued with his deceptive character by telling the parents how the farm had produced high yields. Unfortunately for him, villagers who gathered courage had to face the masquerade and that is how he was caught and killed. This particular type of folktale discourages a life of deception and stealing given the fate that befalls the protagonist. Here one learns about planting and harvesting seasons in an agrarian economy, which necessitates a life of hard work and cooperation rather than deception and stealing.

### 3.2. Parents’ perceptions of the benefits of integrating folktales into school learning tasks

Out of the 15 parents, nine indicated that folktales should be used in schools. Their main reasons were that they believed that folktales were important pedagogical tools for teaching children morality and the ways of their ancestors. These themes were often explicitly stated. For example, one parent said, “I think teaching some lessons using folktales will help the children learn their tradition and those of others.” Another parent added, teachers need to learn some of these folktales which teach people not to steal, fight or kill others. If teachers use these types of folktales in school, children will learn good behavior using interesting methods.

Several parents believed that folktales could teach children important life lessons. For example, one parent explained, “it is very important to introduce folktales in schools especially when teaching about living together as families, sharing with one another. This will help children a lot.” A small number of parents made an explicit connection between use of folktales and pupils’ engagement in learning. For example, one of the parents indicated that folktales should be considered as part of the school program because it will help children to “learn to listen attentively and also share their own stories with others.” Another expanded on the role folktales can play in increasing pupils’ classroom engagement: “folktales should be used to teach lower classes because they are interesting and will make the children not to sleep in class. It will also make them to love school. Any day my daughter’s teacher tells them any folktale in school, she is always very excited and eager to tell us the story. This helps her to recall what was taught in school that day.”

The six parents who did not see the necessity of using folktales in schools, explained their answers in two ways. Some parents focused on the content of folktales and perceived that there was a clear distinction between the subjects for which teachers and family members had responsibility. One of these parents stated, “If teachers want to tell children folktales it should be when they have finished teaching what they are supposed to teach,” while another asked, Of what importance are folktales to children in school? When parents recognized the pedagogical potential of folktales, they often believed that this was relevant only for the informal education provided by families. One of them said, “Parents should narrate their folktales at home while teachers use other methods to teach.”

In summary, 60% of parents perceived that folktales could contribute to improved engagement and learning outcomes for school children and provide an important bridge between formal schooling and the content and pedagogical tools used in children’s informal learning at home and in the wider community. However, the remaining large minority of parents perceived such a bridge to be unnecessary or undesirable.

### 3.3. Teachers’ use of folktales

The results on Table 1 indicate that all the teachers who agreed to participate in the study had used folktales in school. Most teachers used folktales often or very often during both lessons and free time.

**Table 1:** Frequency with which teachers used folktales in two school contexts and the narrator of folktales.

		Percentage of teachers (*n* = 20)	Not at all	Less often	Often	Very often
Context	Lessons	0	5	9	6
	Free time	0	2	6	12
Narrator	Teachers	0	5	8	7
	Pupils	0	7	7	6

Qualitative data indicated that teachers used folktales as a pedagogic tool during lessons that focused on diverse subjects and skills including the composition of text, listening comprehension and moral education. Teachers both narrated folktales themselves and invited their pupils to narrate them (Tables 1, 2). It is noteworthy that no teacher mentioned inviting elders, other cultural experts or parents to tell folktales to their classes. In addition, no teacher mentioned using folktales as an aid in teaching literacy skills. This is likely to be a direct result of the absence of folktales from authorized school textbooks and readers in Cameroon.

**Table 2:** Teachers past and current use of folktales as a pedagogical tool.

Narrator	Exemplar quotations
Teacher	“Most of the time I tell stories when teaching moral education to help the learners understand better…After each lesson I ask questions to the learners to help us come out with the moral lessons” “At times I introduce my composition writing using folk tales. After that, I give guidelines to my pupils to write a composition related to my story” “I use folktales in teaching listening comprehension. At times I tell them it is time for story telling but after that you have questions to answer. Everyone must pay attention”
Pupils	“There are times I prefer to ask children to tell stories when I am teaching. Some start but are unable to complete their folktales” “Some children enjoy narrating folktales to their mates, some narrate the folktales and tell us the moral lesson from the folktales” “I ask the pupils to tell the class any folktale in their local language, then translate to the English language. This is done during Civic education lessons when teaching national languages”

### 3.4. Teachers’ perceptions of the benefits of integrating folktales into school learning tasks

Quantitative data indicated that 17 of the 20 teachers judged that folktales should be incorporated as a pedagogic tool in schools. Although the three other teachers used folktales in their own teaching, two did so to make lessons more interesting for pupils, and didn’t think that it was appropriate for this to be a requirement, since other means of making lessons interesting can also be effective. Despite using folktales in their own practice, one teacher expressed reservations about the use of folktales, indicating that these should only be used for entertainment. Other teachers identified five areas of learning in which folktales had the potential to be a valuable pedagogical tool. In three of these themes, teachers elaborated on their previous responses about the ways they were already using folktales in their role as teacher. Nevertheless, two themes revealed additional uses: helping to strengthen the children’s cultural traditions and extending their repertoire of songs (Table 3).

**Table 3:** Areas of learning in which teachers perceive that folktales have the potential to be a valuable pedagogical tool.

Themes	Subthemes	Some exemplar quotations
Oral transfer of cultural traditions		“I always want children to know their tradition and some of the folktales are based on that” “I use some of the folktales to teach the children the way their forefathers were living…in terms of the traditional way of dressing, medications, food etc” “Teach cultural values”
Cognitive skills	Stimulation of imagination Thinking attention retention	“When children are asked to present folktales in class it enhances their imagination and thinking” “I realized when I asked my pupils to tell stories, in the course of that some will pause for some seconds and will ask me to give them some time to think before they continue” “When it is time for story-telling my pupils pay a lot of attention” “Some children listen to folktales and they also repeat the same stories to their friend”
Morality	Bad behavior is punished Good behavior rewarded Forgiveness	“When am teaching some topics in moral education, I use folktales which show how wicked people are punished” “Folktales can be used to instil knowledge on the type of punishment that can be given to any person who maltreat others, cheat others, kill, tell lies or even those who fight” “I used folktales to show how good people are always rewarded with gifts and other blessings” “There is one famous folktale in the Nso community which focuses on teaching children to help adults, when they do that they will receive blessings” “Folktales are very relevant and should be integrated into the school curriculum to teach good morals. There are many folktales which talk about the importance of forgiving one another”
Language development	New Vocabulary Public speaking Translation skills	“Though I hardly use folktales in teaching, I realized that when I asked my pupils to tell stories, or when I tell them folktales, I asked them to indicate the words that they are getting for the first time. Then we define those words” “Folktales should be encouraged in primary schools because they enhance communication skills in children. They learn to speak in public without any fear. This can help then to become good public speakers or become journalist in future” “Most of the folk tales are often narrated to most children in *lamnso* (the local language of the Nso people). when you ask them to tell stories they translate into the English language’ This helps them in word formation, construction of sentences using diverse tenses, especially the past tense”
Musical repertoire	Learning songs Interest/enjoyment	“I mostly ask learners to tell us the stories. If there is a song in it, they sing together... I tell them to repeat the song severally so that others should learn, the whole story becomes a song to all learners” “Most of my pupils pay attention when the folktale has a song…Some children sing the songs with joy, and this makes the lesson very interesting”

While one teacher indicated that folktales were only valuable during pupils’ free time, all other teachers perceived that folktales are an important and profitable means of promoting learning in elementary school.

## 4. Discussion

The findings suggest that a shared use of folktales as a pedagogic tool has the potential to serve as a bridge between Nso children’s informal learning at home and in their communities and their formal learning in schools. Most Nso parents continue to use folktales in their informal learning practices. They believe that children can learn cultural traditions and values, wisdom and morality from them. They both narrate folktales and invite their children to narrate them. From the findings most parents were very supportive of teachers using folktales in their professional practice either because they valued the content of folktales or because they perceived that they were a useful pedagogical tool for teaching academic subjects. However, some parents, did not perceive that a bridge between the content or pedagogy in children’s home and school learning environments was necessary or desirable. Without an intervention, these parents appear likely to resist any expansion of the use of folktales in schools. Despite this, all the teachers in the current sample used folktales in their professional practice, and most reported that they did so often or very often. Like parents, teachers both narrated folktales and invited pupils to narrate them. The purposes for which they used folktales also overlapped with those reported by parents. Thus, folktales could provide a bridge between home and school learning contexts not only in the form of teaching resources, and the ways in which they are deployed, but in the purposes for which they are used. There was high, but not universal endorsement for folktales to become a standard pedagogic tool in teachers’ professional practice.

Nsamenang (2016) indicates that participatory pedagogies are very important in schools not just for the socialization but for children’s capacity to assume responsibilities for other aspects of learning and development. Folktales can be an important tool for participatory pedagogy. When pupils are asked to narrate folktales and draw lessons from the folktales this instil active participation of the pupils in the learning process. These findings confirmed the work of John Dewey’s pedagogic theory (1966) where emphasis is laid on the importance of learning by doing and development of practical life skills as crucial to children’s education. When learners are given the opportunity to narrate folktales it encourages participatory learning which Dewey advocated.

Morality is one of the key elements of education. From the findings of this study, both teachers and parents indicate that folktales are one of the tools which could be used to inculcate teaching moral lessons to children. The fact that children develop a lot of interest listening or narrating folktales, they can easily internalize the moral values from the stories. Most of the folktales indicate the punishment which comes with negative behavior and the reinforcers which follow positive behavior. Therefore folktales could be integrated into the school program to teach moral education at the elementary level.

## 5. Conclusion

This research was to determine if and how folktales could bridge the divide between Nso children’s learning contexts inside and outside school. The findings indicate that most teachers and parents support the idea of integrating folktales into teachers’ professional practices. They do so because both groups perceive benefits both in the content of folktales (amusement; moral lessons, etc) and in folktales as a pedagogical tool for achieving academic outcomes. These academic outcomes are concentrated in “language arts” for example understanding literary devices like analogy, hyperbole and literacy genres like narrative and song lyrics. It can also be noticed that these folktales enhance the development of skills like classroom engagement, sustained attention, ability to retain and recall information. The fact that most of the teachers often incorporate folktales into their professional practice suggests that there is rich experience and established lesson plans that could be drawn upon in any expansion of the use of folktales by teachers. Although the local community are almost certainly cultural experts in folktales and their performance, none of the teachers reported that they had further strengthened the bridge between learning contexts in and outside school by inviting parents or community members to tell folktales in school and thereby initiating school-community partnerships for learning. If folktales are to be officially integrated into the curriculum where there are accepted, then community experts need to be part of this process. Though it is important to incorporate folktales as a pedagogic tool, this should be done at the elementary levels and in communities where folktales are recognized especially in Africa.

Though this research has created an awareness on the use of folktales in enhancing Nso children’s learning, the sample size was limited to 20 teachers from three primary schools which were all religious and 15 parents in kumbo central sub division in Cameroon. Government schools could not be represented because of the socio-political crisis which led to the shutdown of government schools at that time. It was also difficult to have a representative sample of parents from the schools we were able to recruit from. This is a reality in many low-and middle-income countries where community tension, conflict and disruption are characteristics of the developmental contexts for many children across the world. In order to generalized the findings of this study in Cameroon, it will be necessary for other studies to be carried out in other regions of Cameroon with a representative sample of teachers, parents and children from religious, private and government schools. Since this study is a purely qualitative study it will also be necessary to carry out other studies using mixed methods design.

## Data availability statement

The raw data supporting the conclusions of this article will be made available by the authors, without undue reservation.

## Author contributions

LW conceived the topic, outlined the various phases of the study, reviewed part of the literature which is written in the introduction section, described part of the methodology, conducted some of the interviews for both parents and teachers, took part in the analysis, discussion, and conclusion of the manuscript, and did part of the references. NV reviewed part of the literature on folktales and children’s learning, collected data from some participants, did part of the discussion and the references, and took part in editing the manuscript. All authors contributed to the article and approved the submitted version.

## Conflict of interest

The authors declare that the research was conducted in the absence of any commercial or financial relationships that could be construed as a potential conflict of interest.

## Publisher’s note

All claims expressed in this article are solely those of the authors and do not necessarily represent those of their affiliated organizations, or those of the publisher, the editors and the reviewers. Any product that may be evaluated in this article, or claim that may be made by its manufacturer, is not guaranteed or endorsed by the publisher.
